# Anæsthesia

**Published:** 1872-07

**Authors:** 


### ANÆSTHESIA,

From two Greek words meaning without feeling, is given to such substances as may be taken into the lungs by breathing, or swallowed into the stomach, with the effect of rendering a person insensible to pain and suffering.

OXYGEN GAS

is one of these; for, if taken into the lungs, a skillful dentist can extract several teeth without the person having a particle of pain. Another of these is

ETHER, which seems to have been a discovery of Dr. Horace Wells, a dentist of Hartford, Conn. And as a warning to the reader against the use of this and similar articles as a stimulant or sedative, it may be well to state that Dr. Wells was a portly, noble-looking man, and from seeing the effects of ether, when taken by others, in relieving them from suffering, whether from bodily pain or mental depression, he fell into the habit of taking ether himself; and it grew upon him to such an extent that, being on a visit to the city of New York, and having used it too freely, he became so excited that he was for a while literally beside himself; went into the street after night, and began throwing oil of vitriol on the dresses of the women of the town whenever he met one. For this he was arrested and conveyed to the Tombs. On recovering his senses, he became so disgusted with himself that he resolved on suicide, and saturating his handkerchief with the oblivious ingredient, tied it over his mouth, threw himself back upon his prison cot, and with one tremendous stroke of a razor cut his thigh, cut it to the bone, dividing one of the largest arteries of the body, and died immediately. This was the statement of a personal friend of Dr. Wells, who saw the body a very few moments after the wound was inflicted. Such was the sad fate of a man whose experiments and discovery is one of the greatest boons to humanity. About the same time, Mr. Waldie, a Scotch chemist and bookseller, one day left a saucer half full of chloroform on the floor, and soon after a little dog was lying at the side of it, apparently dead; but after a while began to move one limb, then another, regained consciousness, and frisked away as if nothing was the matter. The chemist, thinking he had made a discovery, administered the chloroform to some cats with a similar result, confirming his belief. He went to Edinburgh, and communicated the facts to Dr. James Simpson, who made some experiments, all proving that breathing the fumes of chloroform had the effect of causing insensibility to pain and suffering, even to the drawing of a tooth or the sawing off a limb. And finally it was ascertained that the most painful surgical operations could be performed without consciousness of any suffering whatever. Prof. Simpson first published these results in 1857. The name given this remarkable agent was terchloride of formyl, which was soon shortened into the present designation,

CHLOROFORM.

Three parts of chlorine gas, and one part of formyl, taking their names thus: chlorine is the Greek name for green, that being the color of the gas, and more appropriate too, from its being largely derived from substances taken from the sea, its plants and salts.

RED ANTS, if made angry, discharge a very pungent acid substance called formic acid, "formica" being the word for ant. If these ants are distilled, a substance is produced so burning, that if it is dropped on the skin, it eats into it like fire. It is also derived from the stinging nettle.

